# Quantitative Cross-Species Extrapolation between Humans and Fish: The Case of the Anti-Depressant Fluoxetine

**DOI:** 10.1371/journal.pone.0110467

**Published:** 2014-10-22

**Authors:** Luigi Margiotta-Casaluci, Stewart F. Owen, Rob I. Cumming, Anna de Polo, Matthew J. Winter, Grace H. Panter, Mariann Rand-Weaver, John P. Sumpter

**Affiliations:** 1 Institute for the Environment, Brunel University, London, United Kingdom; 2 AstraZeneca, Global Environment, Freshwater Quarry, Brixham, United Kingdom; 3 Biosciences, School of Health Sciences and Social Care, Brunel University, London, United Kingdom; Ohio University, United States of America

## Abstract

Fish are an important model for the pharmacological and toxicological characterization of human pharmaceuticals in drug discovery, drug safety assessment and environmental toxicology. However, do fish respond to pharmaceuticals as humans do? To address this question, we provide a novel quantitative cross-species extrapolation approach (qCSE) based on the hypothesis that similar plasma concentrations of pharmaceuticals cause comparable target-mediated effects in both humans and fish at similar level of biological organization (Read-Across Hypothesis). To validate this hypothesis, the behavioural effects of the anti-depressant drug fluoxetine on the fish model fathead minnow (*Pimephales promelas*) were used as test case. Fish were exposed for 28 days to a range of measured water concentrations of fluoxetine (0.1, 1.0, 8.0, 16, 32, 64 µg/L) to produce plasma concentrations below, equal and above the range of Human Therapeutic Plasma Concentrations (H_T_PCs). Fluoxetine and its metabolite, norfluoxetine, were quantified in the plasma of individual fish and linked to behavioural anxiety-related endpoints. The minimum drug plasma concentrations that elicited anxiolytic responses in fish were above the upper value of the H_T_PC range, whereas no effects were observed at plasma concentrations below the H_T_PCs. *In vivo* metabolism of fluoxetine in humans and fish was similar, and displayed bi-phasic concentration-dependent kinetics driven by the auto-inhibitory dynamics and saturation of the enzymes that convert fluoxetine into norfluoxetine. The sensitivity of fish to fluoxetine was not so dissimilar from that of patients affected by general anxiety disorders. These results represent the first direct evidence of measured internal dose response effect of a pharmaceutical in fish, hence validating the Read-Across hypothesis applied to fluoxetine. Overall, this study demonstrates that the qCSE approach, anchored to internal drug concentrations, is a powerful tool to guide the assessment of the sensitivity of fish to pharmaceuticals, and strengthens the translational power of the cross-species extrapolation.

## Introduction

Cross-species extrapolation of biological processes represents a cornerstone in pharmacology and toxicology, both of which are intimately dependent on its reliability to accurately predict therapeutic and harmful effects of chemical substances in humans or other recipient species. However, extrapolating the quantitative relationship between pharmacological or toxicological responses and levels of chemical exposure, from one species to another, remains one of the key challenges for the development of high-power and low-risk predictive approaches [1], [2]. Classically, the extrapolation is applied from the model species (e.g. rodents) to humans using appropriate scaling factors (e.g. safety margins) that take into account inter-species differences in metabolism, physiology, genetics, and biochemistry [3]. For example, animal experiments are used to estimate Acceptable Daily Intake (ADI) values for chemicals in food and drinking water [4], [5], and to perform the pre-clinical safety assessment of pharmaceuticals [6]. On the other hand, when the extrapolation is aimed at the environmental risk assessment (ERA) of chemicals applied to wildlife (e.g. aquatic organisms) the results of toxicity experiments, conducted using a limited selection of test species, are extrapolated to tens or hundreds of species potentially living within the same ecosystem [7].

In the last two decades, the field of ERA has been posing a conceptual challenge to the cross-species extrapolation process, due to presence of human pharmaceuticals in the aquatic environment [8]–[11]. The increasing number of pharmaceuticals detected in surface waters (e.g. rivers) has attracted interest in recent years because these compounds, despite being present at very low concentrations (sub-ng/L to few µg/L), are selected to interact with high affinity with specific human biological targets (e.g. receptors, enzymes). These targets can be evolutionary conserved and functional also in aquatic organisms (especially in fish) [12], suggesting that the interaction drug/target may theoretically lead to unwanted pharmacological (and potentially toxicological) effects in non-target species exposed to pharmaceuticals in the environment. This potential scenario, combined with the fact that for many pharmaceuticals there are vast amounts of data generated on their pharmacology and toxicology during and after the development phase [13], led to the development of an alternative extrapolation framework, known as “Read-Across” [11]. This approach centres on the exploitation of clinical and non-clinical data to predict potential effects in wildlife species, and it has been praised by a number of authors over recent years, particularly with respect to fish [11], [13]–[15].

Although this approach in its present form is largely theoretical, supporting experimental data are emerging to suggest that Read-Across, coupled with bioinformatics and with the use of biological databases, could become one of the most promising approaches to predict potential adverse effects induced by pharmaceuticals in non-target species [11], [16], [17]. Nonetheless, the Read-Across approach in its present formulation is mainly qualitative, whereas qualitative extrapolations between species are not robust enough to drive decision-making, allow the generation of inter-species safety margins for drug safety assessment or provide quantitative indicators of environmental risk.

Here we provide a novel quantitative cross-species extrapolation approach (qCSE) based on the Read-Across hypothesis [11], [18]. Assuming the evolutionary and functional conservation of molecular targets in fish, the hypothesis states that human pharmaceuticals will elicit the same target-mediated pharmacological response in fish as they do in humans, that the pharmacological responses will precede the toxicological ones, and that target-mediated effects at a comparable level of biological organization will occur at similar plasma (or tissue) concentrations (i.e. Human Therapeutic Plasma Concentration, H_T_PC) [11]. According to this hypothesis, it should be possible to use the relationship between internal concentrations (either measured or predicted) in the target (i.e. human) and non-target (i.e. fish) organisms to predict the likelihood of an effect occurring.

Currently no comprehensive validation of the Read-Across Hypothesis exists [11]; therefore, in our study, the behavioural effects of the anti-depressant drug fluoxetine on the fish model fathead minnow (*Pimephales promelas*) were used as test case. Fluoxetine is a Selective Serotonin Reuptake Inhibitor (SSRI) anti-depressant used globally for over 25 years to treat major depression and other psychiatric disorders, and which has been frequently detected at low concentrations in the aquatic environment [19]. Critically, the primary pharmacological target, the serotonin transporter, is evolutionary and functionally conserved in fish [20], and fluoxetine has previously been reported to affect fish behaviour [21], [22]. In particular, as SSRIs are recognised anxiolytics used to treat Generalised Anxiety Disorder (GAD) in humans [23], [24] and anxiogenic/anxiolytic responses are well characterized in adult fish [25], [26]. Anxiety-related behaviour was selected as an appropriate endpoint in order to test and compare Mode-of-Action (MoA) driven effects between target and non-target species [21]. In particular, fluoxetine-induced anxiolytic responses in fish were hypothesised as functionally equivalent to the reduction of anxiety in human patients following fluoxetine treatment.

We exposed fish to a range of water concentrations of fluoxetine predicted to produce plasma concentrations below, equal and above the ones known to induce therapeutic effects in humans (i.e. H_T_PC). We then measured the drug plasma concentrations in individual fish and linked them to anxiety-related endpoints quantified using automated video-tracking software. The aim of the study was to assess whether behavioural responses were induced by fluoxetine at plasma concentrations higher, equal or lower than H_T_PCs. Our results demonstrated that fluoxetine induces behavioural effects in fish as it does in humans, but only when its blood levels are similar to those effective in patients, hence validating the Read-Across hypothesis. Overall we show that the qCSE approach, anchored to the plasma concentration of drug, is a powerful tool to guide the assessment of the sensitivity of fish to pharmaceuticals, and strengthens the translational power of the cross-species extrapolation.

## Materials and Methods

### Ethics statement

This study was carried out at AstraZeneca (UK) under Project License and Personnel Licences granted by the UK Home Office under the United Kingdom Animals Act (Scientific Procedures), and also in accordance with AstraZeneca’s local and global ethical policies.

### Test species

Adult male fathead minnows (*Pimephales promelas*), approximately 6 months old (weight: 2.9±1 g) were supplied from stocks maintained at AstraZeneca and kept according to the UK Animal Scientific Procedures Act guidelines.

### Test chemical and dilution water

Fluoxetine hydrochloride (CAS number 56296-78-7), was obtained from VWR (supplier US Pharmacopeia) as >99% pure. Concentrated stock solutions were prepared weekly in reverse osmosis water. Dechlorinated tap water (5 and 10 µm carbon filtered) was used as dilution water. Temperature was maintained at 25±1°C for the duration of the study. Dissolved oxygen concentrations remained above 80% of the air saturation value throughout the exposures and the *p*H was 7.5±0.5. The hardness of the dilution water was <200 mg L^−1^, as CaCO_3_, and residual chlorine was <0.01 mg L^−1^.

### Experimental design and exposure protocol

The 28-day experiment was carried out using a continuous flow-through system comprised of 9.5 L glass tanks. Thermostatically heated (25±1°C) dechlorinated tap water flowed into eight glass mixing chambers at a rate of 333.3 mL/min. The same chambers also received the stock solution of the test chemical via peristaltic pump at a rate of 0.1 mL/min, in order to achieve the desired nominal concentrations. Separate glass lines from each mixing chamber supplied about 12 tank volume changes per day to each of four replicate test tanks, each containing five male fish (20 fish per treatment).

The final experimental setup included eight treatment groups. Due to the high variability of behavioural responses, two independent sets of dilution water control groups were used (C1 and C2) in order to increase the statistical power of the experiment. Moreover, six different fish groups were exposed to six concentrations of fluoxetine (0.1, 1.0, 8.0, 16, 32, 64 µg/L), selected to cover both environmentally-relevant concentrations and pharmacologically relevant ones. The selection of test water concentrations was also driven by the application of the Fish Plasma Model [18] (as detailed in the following section), which predicted resulting fish plasma concentrations respectively below (groups: 0.1, 1.0, 8.0 µg/L), within (groups: 8.0, 16 µg/L), and above (groups: 32, 64 µg/L) the Human Therapeutic Range of fluoxetine. The latter was generated by a clinical trial in which patients were treated for 30 days with daily administration of fluoxetine (40 mg). This dosing regimen produced plasma concentrations of fluoxetine in the range 91–302 ng/mL and norfluoxetine in the range 72–258 ng/mL (FDA, Application No. 18-936/SE5-064). The duration of the clinical trial (30-days) was very close to the duration of the fish exposure experiment described here (28 days).

Both the allocation of the treatment groups in the experimental room and the allocation of each fish into one of the 32 tanks were performed randomly using random number generator software. Due to the large number of fish and treatments, and to the time required to perform the subsequent behavioural tests, the allocation of the fish to the experimental tanks was performed over two days, as was the final sampling. During the study, fish were fed three times per day, once with adult brine shrimp (Tropical Marine Centre, Gamma irradiated) and twice with pellet food (Bio-Optimal C80, Brande, Denmark). The test was run at 25±1°C, with 16 h light/8 h dark photoperiod, and with 20 min dawn/dusk transition periods.

### Prediction of fluoxetine plasma concentrations by the Fish Plasma Model

Measured plasma concentrations were compared to the concentrations predicted by the Fish Plasma Model. The model aims to predict the Fish Steady State Plasma Concentration (F_SS_PC) of a drug starting from a given water concentration, and is based on the following equations:
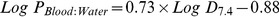
(1)

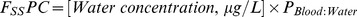
(2)


In the original model equation (2) includes the Log K_OW_ instead of the Log D_7.4_. However, Valenti and collaborators [27] demonstrated that water pH significantly affects the ionization, bioavailability, and toxicity of SSRIs. For this reason, fluoxetine Log D_7.4_ (1.99; [28]) was used to run the model instead of the Log K_OW_ (4.09; predicted by ALOGPS).

The nominal water concentrations of fluoxetine were used in Equation (2) to drive the selection of the exposure concentration; whereas the water concentrations measured on Day 28 were used to compare measured *versus* predicted plasma concentrations.

### Analysis of fish behaviour

#### Methodological rationale for the selection of the Novel Tank Diving Test

**Figure 1:**
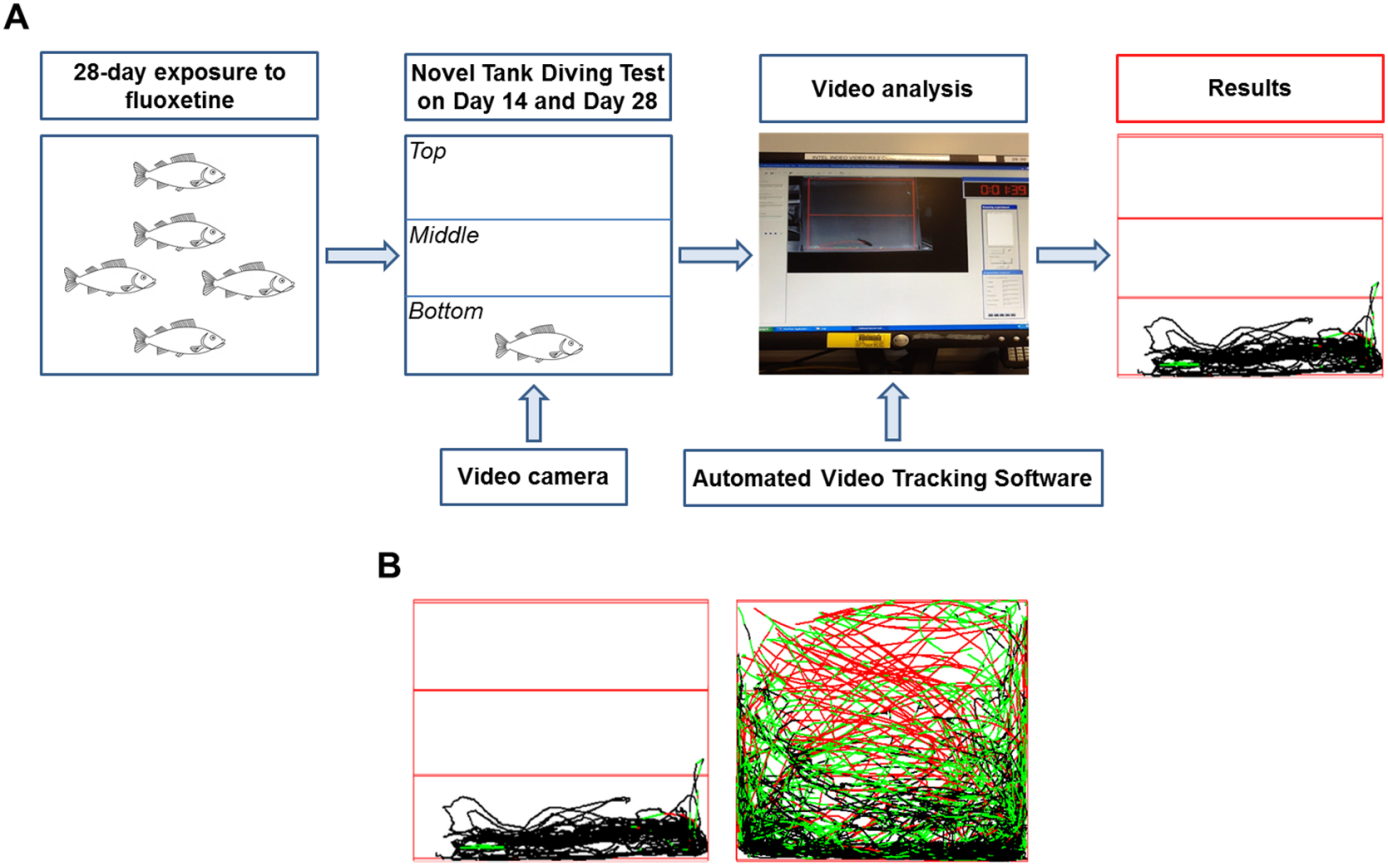
Methodological procedure for the quantification of fish behaviour. A) Experimental steps performed to quantify fish behavioural response to a new environment following 14-day and 28-day exposure to fluoxetine. Anxiety-related behavioural endpoints were quantified using a Novel Tank Diving Test. B) Example of different exploratory behaviours in a Novel Tank Diving Test. Inactive fish (left) *versus* active fish (right). The different tracking colours indicate different speeds (black, slow; green, medium; red, fast).

The “Novel Tank Diving Test” is conceptually similar to the rodent open field test. It is based on the instinctive behaviour of fish to seek protection when they are transferred to a novel and unfamiliar environment (i.e. observation tank) by diving to the bottom and remaining in an alert status until the environmental conditions are perceived as safe enough to initiate exploration of the new environment. In this test, fish are individually transferred from the home tank to the novel environment, which is represented by a clean observation tank containing water that has not previously been exposed to other fish. The swimming pattern is then analysed by separating the main area into two or three different sub-areas (e.g. bottom, middle, top) in post hoc video analysis (Figure 1). Several behavioural endpoints are quantified to assess anxiety (e.g. number of transitions into the top area, time spent in the top area). Many of these endpoints are strictly correlated with the height at which the fish swims in the tank (Figure 1). This measure is considered a very powerful measure of anxiety, and notably, it can be compared to the thymogtaxis observed in rodents, defined as the response of an organism to physical contact or to the proximity of a physical discontinuity in the environment (e.g. rats preferring to swim near the edge of a water maze) [29].

#### Novel Tank Diving Test and Automated video-tracking of fathead minnow behavior

The behavioural test apparatus consisted of five glass observation tanks (305×355×210 mm; L×H×D) filled with 10.0 L of water. All the sides of the tanks, except the observational one, were covered with white paper in order to enhance the contrast between fish and background, to facilitate the software video analysis, and to avoid visual disturbance to the fish. One Fujifilm Digital Camera (FinePix JV300, 14.0 Mpix) was positioned in front of each observation tank.

On Day 28, each behavioural trial started with the random selection of one of the experimental tanks. The only exception to the random selection was represented by the test of one control group at the beginning of the day (i.e. C1) and one at the end (i.e. C2). This was necessary in order to exclude the time of the day as a source of difference in fish behavioural responses (e.g. fish tested at 9 am *vs* the ones tested at 5 pm). Then the water in the observation tanks was spiked with the appropriate volume of fluoxetine stock solution in order to reach a fluoxetine concentration equal to the one present in the selected exposure tank, thus avoiding potential loss of drug from fish blood during the duration of the behavioural test. All the five fish in the tank were gently collected and individually allocated to one of the five observation tanks. Fish behaviour in response to the novel environment was recorded for 20 minutes, and was initiated within 30 seconds from the allocation of the last fish.

At the end of the trial, fish were quickly removed from the observation tanks, and terminally anaesthetised according to UK Home Office regulations using a buffered ethyl 3-aminobenzoate methanesulfonate solution (MS-222, 0.5 g/L; pH 7.5) (Sigma, Poole, UK; CAS No: 144-55-8). Immediately after termination, wet weight (to the nearest 0.0001 g) and standard length (to the nearest 0.1 mm) were recorded, and these parameters were used to calculate the condition index (CI). Blood samples were collected from each fish via the caudal artery/vein using 75 µL heparinised haematocrit capillary tubes, which were subsequently centrifuged at 14,000×g for 5 min at 4°C in order to determine the haematocrit fraction and to separate the plasma fraction. The latter was collected (typically between 10 and 50 µL) and individually transferred to a 96-well plate for the analytical quantification of fluoxetine and norfluoxetine. Lastly, the brain and liver were rapidly dissected, weighed (to the nearest 0.0001 g), and snap frozen in liquid nitrogen before being stored at −80°C. The liver weight was used to calculate the hepatosomatic index (HSI). The sequence described above was performed by a team of operators working in parallel. This guaranteed that all the blood samples were collected within three minutes from the fish termination, thus minimizing any potential metabolic alteration of the concentration of fluoxetine and of its metabolite, norfluoxetine.

At the end of each trial on a group of 5 fish, the water in the observation tanks was discarded, the tanks were washed thoroughly using clean water, and clean water (10 L) was dispensed in each tank. This precaution was adopted in order to avoid any potential chemical communication between the tested fish and the following one occurring through the release of alarm pheromones into the water, or through faeces. The next trial was then initiated, repeating the entire procedure described above.

The VideoTrack analysis software (Version 2.5.0.25, ViewPoint, Lyon, France) was used for the offline analysis of fish exploratory behaviour. The observation tank was visually divided in three areas of equal size (bottom, middle, top), and the following seven endpoints were quantified for a period of 20 min: number of entries into the top area (*T-Top*), number of entries into the middle area (*T-Middle*), percentage of time spent in the top area (*Time-Top*), percentage of time spent in the middle area (*Time-Middle*), distance travelled in the top area (*Dist-Top*), distance travelled in the middle area (*Dist-Middle*), total distance travelled (*Dist-Tot*), and swim velocity (*Speed*).

As well as assessing behaviour at the end of the experiment, the same behavioural test was performed on Day 14 on two out of four replicate tanks per treatment group (n = 10), with the exception that fish were not terminated after the test, but were reassigned to the original exposure tank. The tested tanks were selected by using a random number generator.

#### Weekly behavioural observations

To investigate whether the geotaxis response observed during the Novel Tank Diving Test was also detectable in the exposure tanks, an operator performed a semi-quantitative and blind analysis of group behaviour on videos recorded in each exposure tank. On Day 0, 6, 12, 18, 24 fish were recorded for 25 min in their exposure tank using Fujifilm Digital Camera (FinePix JV300, 14.0 Mpix) positioned in front of the tanks. The video-recording were performed one hour after feeding, twice a day at 1 pm and 7 pm. Since no intra-day differences in the group behaviour were observed, only the videos recorded at 1 pm were analysed blindly by an independent operator not involved in the research project. The analysis was performed for 12 min, from minute 10 to minute 22 of each video, and consisted in recording the number of seconds during which a minimum of three fish out of five were in the upper area of the exposure tank. This endpoint was selected after the qualitative observation, in a previous experiment, that fish treated with 100 µg fluoxetine/L spent more time swimming in the upper part of the tank than control fish.

### Quantification of fluoxetine in water

Water samples (5–10 mL) were collected from each fish tank on day -1, 1, 7, 14, 21 and 28. Samples were diluted with an equal volume of acetonitrile (ACN) containing 2.0 µg/L of the internal standards: fluoxetine-d5 and norfluoxetine-d5. Samples were analysed by liquid chromatography-mass spectrometry (LC-MS) and quantified against known concentrations of fluoxetine-d5 and norfluoxetine-d5. The details of the LC-MS apparatus and protocol are provided in Table S1 and Table S2.

### Quantification of fluoxetine and norfluoxetine in fish plasma

Fish plasma samples (5 to 50 µL) were transferred to 96-deepwell plate and 400 µL of ACN was added to each well. Successively, samples were extracted by solid phase extraction (SPE) using a Strata-X-C plate (Strata-X-C, 96-well, 30 mg, Phenomenex, Torrance, California). Each plasma sample was loaded to the SPE plate preconditioned with methanol and equilibrated with ACN. The compounds were eluted with 1 mL 5% ammonium hydroxide in CAN/i-propanol solution (50/50, v/v). The elutes were collected in 1 mL plastic vials in a 96-well plate array and evaporated to dryness under vacuum at 30°C. Samples were reconstituted in 500 µL of ACN containing 2.0 µg/L of the internal standards and 500 µL of HPLC water. Samples were then analysed by LC-MS and quantified against known concentrations of fluoxetine-d5 and norfluoxetine-d5. The details of the LC-MS apparatus and protocol are provided in Table S1 and Table S2. Spiked control plasma samples (20 µg/L) were prepared in triplicate and were used to assess the extraction efficiency.

### Statistical analysis

Statistical analyses were conducted using SPSS software (version 20, SPSS Inc, USA). Data were analysed for normality (Kolmogorov–Smirnov test) and variance homogeneity (Levene’s test). Where assumptions of normality and homogeneity were met, one-way analysis of variance (ANOVA) was followed by the Dunnett’s test to compare the treatment means with controls. Where the assumptions were not met, data were transformed ((log(x+1)), and analysed using a non-parametric test, Kruskal–Wallis ANOVA on Ranks, followed by Dunn’s post hoc test, and by Wilcoxon-Rank-Sum Test [30]. Statistical significance was set at a level of *p*<0.05, unless otherwise indicated.

## Results

### Water concentrations of fluoxetine

Concentrations of fluoxetine in water were in the expected range for all treatment groups. Mean measured concentrations in the 1, 8, 16, 32 and 64 µg/L groups were, respectively, 1.2±0.3, 9.1±1.3, 17.4±2.2, 41.7±8.0 and 72.5±9.3 µg/L. Fluoxetine concentration in the 0.1 µg/L treatment group was below the LOD (0.4 µg/L); however, the concentration of the dosing stock used in the 0.1 µg/L treatment group was quantified on two occasions during the study and was 100% of the nominal concentration. Since the measured flow rates of the dosing stocks and dilution water were equal to the nominal values for all the treatments, it is assumed that the nominal water concentration was also achieved in the 0.1 µg/L group. Concentrations of fluoxetine in the control groups (C1 and C2) were below the LOD. Finally, norfluoxetine concentrations were below the LOD (0.8 µg/L) in all the water samples analysed.

Due to the importance of water concentrations to determine the plasma concentration by the FPM, the water concentrations measured on the sampling day (Day 28) will be used in this manuscript. These values were 0.1, 1.1, 9.7, 19.6, 38.4, and 72.2 µg fluoxetine/L and are approximated to the nearest integer (0.1, 1, 10, 20, 38, and 72 µg fluoxetine/L).

### Fluoxetine uptake and metabolism

Fluoxetine was detected in fish plasma and was shown to be transformed to the metabolite norfluoxetine. Fluoxetine and norfluoxetine were successfully quantified in individual plasma samples, allowing the assessment of the inter-individual variability of drug uptake and its relationship with the biological effects. The two target compounds were detected in all the fish, except those exposed to 0.1 and 1 µg/L, in which the concentrations of the two analytes were below the LOD and LOQ of the analytical method.

**Figure 2:**
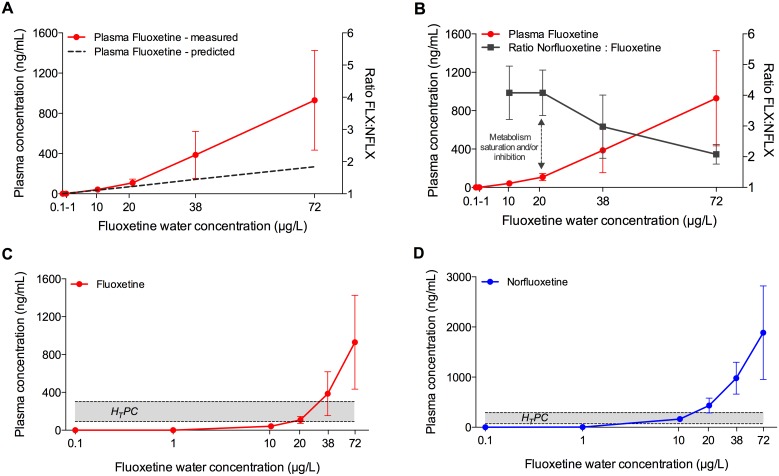
Fluoxetine uptake and metabolism in fathead minnow following a 28-d study. A) Relationship between measured (red line; mean ± SD; n = 20) and predicted (dashed line) plasma concentrations of fluoxetine, based on concentrations quantified in the water. The predicted plasma concentrations were generated by using the Fish Plasma Model [18]. B) Relationship between measured plasma concentrations of fluoxetine (red line; mean ± SD; n = 20) and norfluoxetine:fluoxetine ratio (grey line, mean ± SD; n = 20). The change in the slope of the plasma concentration curve corresponds to the decrease of the norfluoxetine:fluoxetine ratio, indicating inhibitory and/or saturation effects on the metabolic enzymes that convert fluoxetine into norfluoxetine. C) Relationship between measured plasma concentrations of fluoxetine in fish plasma (mean ± SD; n = 20) and Human Therapeutic Plasma Concentration range (grey area, 91–302 ng/mL). D) Relationship between measured plasma concentrations of norfluoxetine in fish plasma (mean ± SD; n = 20) and Human Therapeutic Plasma Concentration range (grey area, 72–258 ng/mL).

The uptake followed a bi-phasic concentration-dependent kinetics (Figure 2). The lowest fluoxetine water concentrations that produced quantifiable levels of fluoxetine and norlfuoxetine in individual plasma samples was 10 µg fluoxetine/L. In this treatment group the measured plasma concentrations were very close to those predicted by the FPM, which was therefore also used to predict plasma concentrations for the 0.1 and 1 µg/L treatment groups. At water concentrations between 0.1 and 20 µg/L the linear uptake was driven by the Log D_7.4_, as demonstrated by the high accuracy of the FPM. However, at water concentrations higher than 20 µg/L, the slope of the linear uptake changed significantly (from 4.3 to 15.5), so that the measured plasma concentrations were higher than those predicted. At 10 µg/L the mean measured plasma concentrations were only 14% higher than the predicted ones. This value increased to 32% at 20 µg/L, 63% at 38 µg/L, and 71% at 72 µg/L.

The linear equation (R^2^ = 0.9992) describing the plasma uptake of fluoxetine at water concentrations between 20 and 72 µg/L is the following:
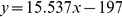
(3)where “x” is the water concentration and “y” the plasma concentration.

The described change in the slope of the uptake curve was concomitant to the change in the ratio between norfluoxetine and fluoxetine (NFLX:FLX) (Figure 2). At fluoxetine plasma concentrations between 0 and 100 ng/mL, the ratio NFLX:FLX was 4∶1; whereas above plasma concentrations of 100 ng/mL the ratio was increasingly lower at increasing plasma concentrations. The lowest NFLX:FLX value observed in this study was 2∶1 and corresponded to a plasma concentration of approximately 900 ng/mL.

### Fish Plasma Concentrations *vs* Human Therapeutic Plasma Concentrations

The H_T_PC range used as reference in this study was 91–302 ng/mL for fluoxetine and 72–258 ng/mL for norfluoxetine (FDA, Application No. 18-936/SE5-064).

Exposure of fish to 0.1, 1 and 10 µg/L resulted in plasma concentrations of fluoxetine below the H_T_PC, whereas exposure to 20 and 38 µg fluoxetine/L produced plasma concentrations of fluoxetine approximately equal to the lower and the higher values of the H_T_PC range, respectively (Figure 2). On the other hand, exposure to the highest concentration of fluoxetine (72 µg/L) resulted in plasma concentrations between 3- and 10-fold above the higher and the lower value of the H_T_PC range, respectively. The mean plasma concentration of fluoxetine in those fish (930 ng/mL) was just below the plasma concentration defined as “toxic” in humans (1000 ng/mL) [31]. However, four fish out of twenty exceeded that value without showing any evident symptom of toxicity (2632, 1664, 1052, 1037 ng/mL).

When norfluoxetine was considered, the relationship with the H_T_PCs reflected the apparently higher metabolic capacity of fish compared to humans in metabolising fluoxetine to norfluoxetine. Indeed, differently from plasma fluoxetine, exposure to 10 µg fluoxetine/L was sufficient to produce plasma concentrations of norfluoxetine within the H_T_PC range. At the same time, exposure to 20, 38 and 72 µg fluoxetine/L produce plasma concentrations of norfluoxetine, respectively, 1.7-, 3.8-, and 7.3–fold above the highest value of the H_T_PC range (258 ng/mL) (Figure 2).

Since in humans fluoxetine and norfluoxetine are considered equipotent, the sum of the two compounds it is what should be considered in order to properly interpret the biological effects. Here we made the assumption that fluoxetine and nor-fluoxetine are also equi-potent in fish. In doing so, it was possible to observe that exposure to 0.1 and 1 µg fluoxetine/L resulted in plasma concentrations of the two compounds that were below the lowest value of the H_T_PC range. On the other hand, fish exposed to 10 and 20 µg/L had total plasma concentrations of the two compounds very similar to, respectively, the lowest and the highest value of the H_T_PC range (163 *vs* 208 ng/mL and 541 *vs* 560 ng/mL). Lastly, the two top exposure concentrations corresponded to plasma levels respectively 2.4 and 5-fold above the highest value of the H_T_PC range.

### Intra-group variability of drug uptake

The maximum observed inter-individual variability of plasma concentrations in fish kept in the same exposure tank was 7.6-fold difference between the minimum and the maximum measured values for fluoxetine (Table 1), and 4-fold difference for norfluoxetine (Table 2). When the four replicates per treatment were combined, these values increase to 8.6-fold for fluoxetine (Table 1), and 5.3-fold for norfluoxetine (Table 2). However, the minor differences in the water concentration of fluoxetine among the four replicate tanks during the study may account for this apparent increase in variability. In most tanks, inter-individual variability of drug plasma concentrations was consistently lower than 3-fold [i.e. 11 out of 16 tanks for fluoxetine, and 15 out of 16 tanks for norfluoxetine]. There was no apparent water concentration-dependent increase of variability for fluoxetine, whereas an increasing trend can be observed for norfluoxetine. However, the latter was closely related to inter-individual variability in fish metabolism rather than to uptake processes.

**Table 1:** Inter-individual and intra-treatment variability of fluoxetine plasma concentrations.

Treatment	Samplesize	Mean	Median	Min	Max	RatioMax:Min	25%	75%
***10*** ***µg/L – Rep A***	5	48.4	37.4	31.8	72.2	2.3	34.6	66.8
***10*** ***µg/L – Rep B***	5	46.4	44.0	21.1	84.7	4.0	33.8	54.3
***10*** ***µg/L – Rep C***	5	39.3	41.0	29.5	45.8	1.6	35.5	43.3
***10*** ***µg/L – Rep D***	4	31.7	27.4	24.5	47.6	1.9	24.7	38.8
***10*** ***µg/L – ALL FISH***	19	42.0	38.1	21.1	84.7	4.0	30.4	45.4
***20*** ***µg/L – Rep A***	5	105.1	97.9	64.9	147.4	2.3	86.9	127.5
***20*** ***µg/L – Rep B***	5	95.2	107.9	53.0	129.0	2.4	71.3	113.9
***20*** ***µg/L – Rep C***	5	95.5	84.9	59.1	158.5	2.7	70.2	115.3
***20*** ***µg/L – Rep D***	5	136.8	128.3	90.3	183.0	2.0	98.3	181.8
***20*** ***µg/L – ALL FISH***	20	108.1	100.9	53.0	183.0	3.5	81.1	128.7
***38*** ***µg/L – Rep A***	5	256.3	283.0	104.3	385.9	3.7	167.0	337.0
***38*** ***µg/L – Rep B***	3	451.7	342.8	265.9	746.3	2.8	285.1	645.4
***38*** ***µg/L – Rep C***	5	435.9	338.1	223.9	892.6	4.0	255.7	567.1
***38*** ***µg/L – Rep D***	5	428.0	391.6	116.3	881.4	7.6	235.6	577.0
***38*** ***µg/L – ALL FISH***	18	386.5	329.4	104.3	892.6	8.6	265.9	458.6
***72*** ***µg/L – Rep A***	5	700.4	723.7	645.3	728.5	1.1	669.1	727.7
***72*** ***µg/L – Rep B***	4	1249.7	867.4	632.0	2632.1	4.2	745.2	1754.3
***72*** ***µg/L – Rep C***	5	944.2	946.3	779.0	1051.7	1.3	875.2	1040.6
***72*** ***µg/L – Rep D***	4	877.6	659.4	527.9	1663.6	3.2	541.2	1214.0
***72*** ***µg/L – ALL FISH***	18	929.6	771.7	527.9	2632.1	5.0	677.0	946.3

The unit of the values indicated in the columns Mean, Median, Min, Max, 25%, and 75% is ng/mL.

**Table 2:** Inter-individual and intra-treatment variability of norfluoxetine plasma concentrations.

Treatment	Samplesize	Mean	Median	Min	Max	RatioMax:Min	25%	75%
***10*** ***µg/L – Rep A***	5	189.7	215.2	98.4	272.1	2.8	122.1	242.6
***10*** ***µg/L – Rep B***	5	157.5	129.1	92.9	255.7	2.8	117.7	201.8
***10*** ***µg/L – Rep C***	5	167.4	173.5	98.5	214.6	2.2	150.8	190.2
***10*** ***µg/L – Rep D***	4	134.7	126.1	102.5	184.2	1.8	107.9	161.5
***10*** ***µg/L – ALL FISH***	19	163.8	168.3	92.9	272.1	2.9	116.6	207.0
***20*** ***µg/L – Rep A***	5	444.3	420.4	322.9	575.7	1.8	355.9	545.5
***20*** ***µg/L – Rep B***	5	378.9	436.8	190.1	490.0	2.6	281.1	472.1
***20*** ***µg/L – Rep C***	5	363.9	387.3	261.7	458.8	1.8	282.1	431.7
***20*** ***µg/L – Rep D***	5	544.4	477.8	349.6	869.2	2.5	384.8	689.0
***20*** ***µg/L – ALL FISH***	20	387.7	403.9	190.1	575.7	3.0	300.2	458.8
***38*** ***µg/L – Rep A***	5	788.1	847.1	462.9	930.6	2.0	721.8	901.6
***38*** ***µg/L – Rep B***	3	1127.8	1267.1	811.2	1305.1	1.6	925.2	1295.6
***38*** ***µg/L – Rep C***	5	883.9	773.7	487.8	1265.6	2.6	687.1	1170.7
***38*** ***µg/L – Rep D***	5	1176.4	1250.6	620.4	1627.1	2.6	948.1	1401.7
***38*** ***µg/L – ALL FISH***	18	979.2	911.2	462.9	1627.1	3.5	773.7	1265.6
***72*** ***µg/L – Rep A***	5	1528.4	1691.0	998.1	1805.1	1.8	1317.1	1744.6
***72*** ***µg/L – Rep B***	4	2556.8	1812.2	1328.2	5274.6	4.0	1461.0	3652.7
***72*** ***µg/L – Rep C***	5	1878.2	1811.8	1494.7	2419.3	1.6	1569.3	2158.2
***72*** ***µg/L – Rep D***	4	1674.3	1619.7	1003.1	2454.5	2.4	1237.2	2111.4
***72*** ***µg/L – ALL FISH***	18	1886.5	1707.7	998.1	5274.6	5.3	1471.2	2030.8

The unit of the values indicated in the columns Mean, Median, Min, Max, 25%, and 75% is ng/mL.

### Behavioural effects

The following seven endpoints were quantified for a period of 20 minutes: number of entries into the top area (*T-Top*); number of entries into the middle area (*T-Middle*); percentage of time spent in the top area (*Time-Top*); percentage of time spent in the middle area (*Time-Middle*); distance travelled in the top area (*Dist-Top*); distance travelled in the middle area (*Dist-Middle*); total distance travelled (*Dist-Tot*), and swim velocity (*Speed*).

#### Novel Tank Diving Test – Day 14

**Figure 3:**
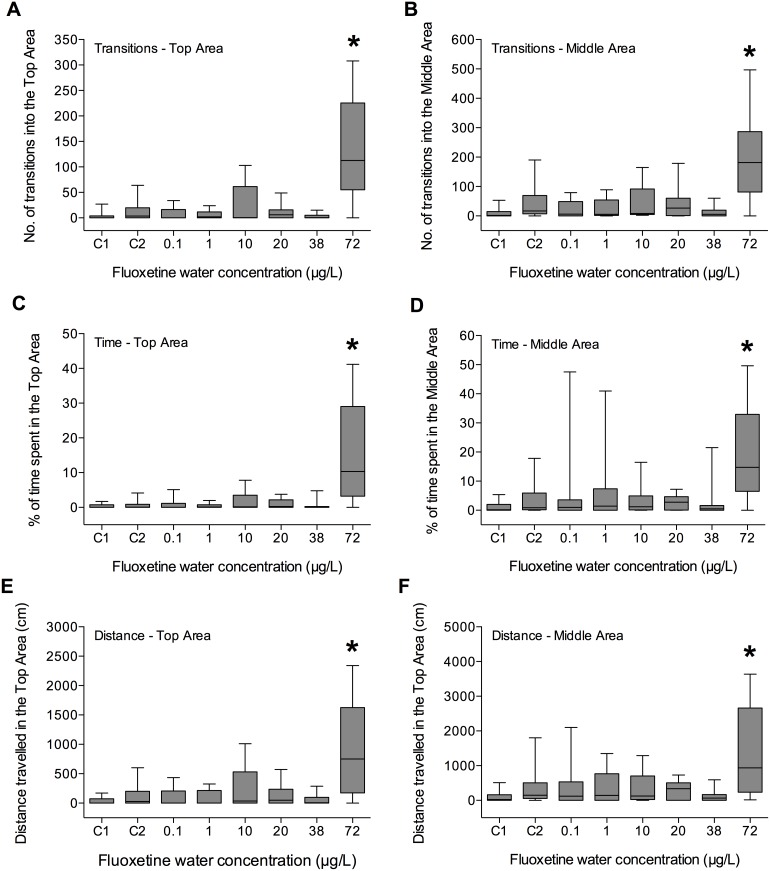
Effect of fluoxetine on fish exploratory behaviour quantified during a Novel Tank Diving Test performed after 14 days of exposure. A) Number of transitions into the Top Area; B) number of transitions into the Middle Area; C) time spent in the Top Area; D) time spent in the Middle Area; E) distance travelled in the Top Area; F) distance travelled in the Middle Area. C1 and C2 indicate control group 1 and control group 2, respectively. Boxes represent medians (full line), with 5th and 95th percentiles (*n* = 20). *p<0.05.

After 14 Days of exposure to fluoxetine, only fish exposed to the highest concentration (72 µg/L) showed a significantly enhanced exploratory behaviour of the novel environment. All the endpoints, except *Speed*, were significantly different from the control values (*p*<0.01) (Figure 3).

#### Novel Tank Diving Test – Day 28

**Figure 4:**
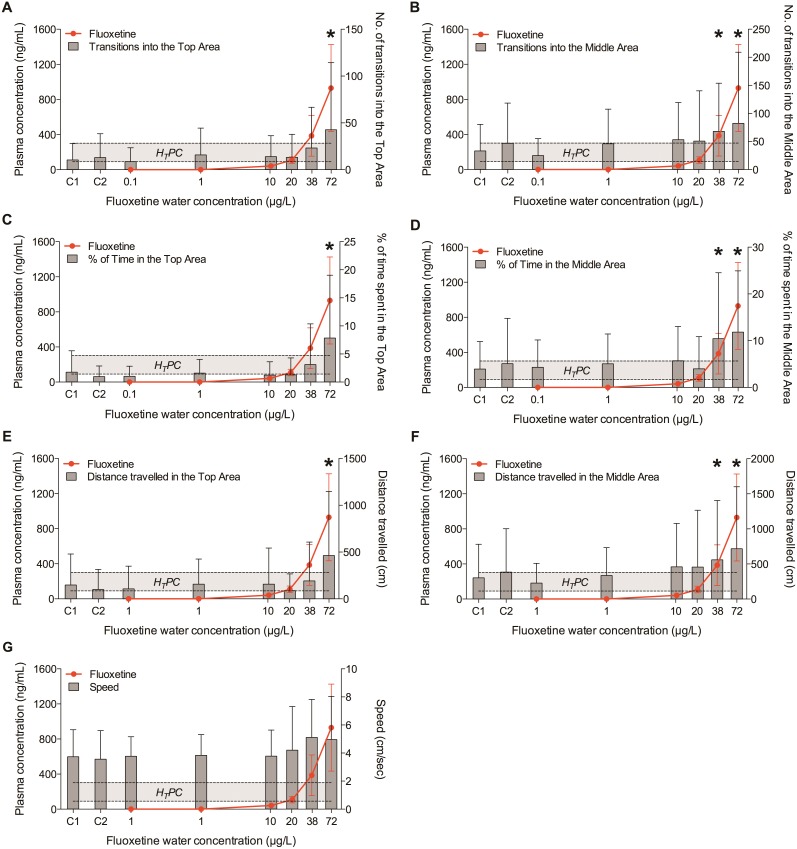
Relationship between plasma concentrations of fluoxetine and its effects on fish exploratory behaviour after 28 days of exposure. Exploratory behaviour was quantified in individual fish using the Novel Tank Diving Test. A) Number of transitions into the Top Area; B) number of transitions into the Middle Area; C) time spent in the Top Area; D) time spent in the Middle Area; E) distance travelled in the Top Area; F) distance travelled in the Middle Area; G) speed. The Human Therapeutic Plasma Concentration range of fluoxetine plotted in the graphs is 91–302 ng/mL. C1 and C2 indicate control group 1 and control group 2, respectively. The X-axis has a Log2 scale, while the Y-axis has a linear scale. Values are plotted as mean ± SD (*n* = 20). *: *p*<0.05.

**Figure 5:**
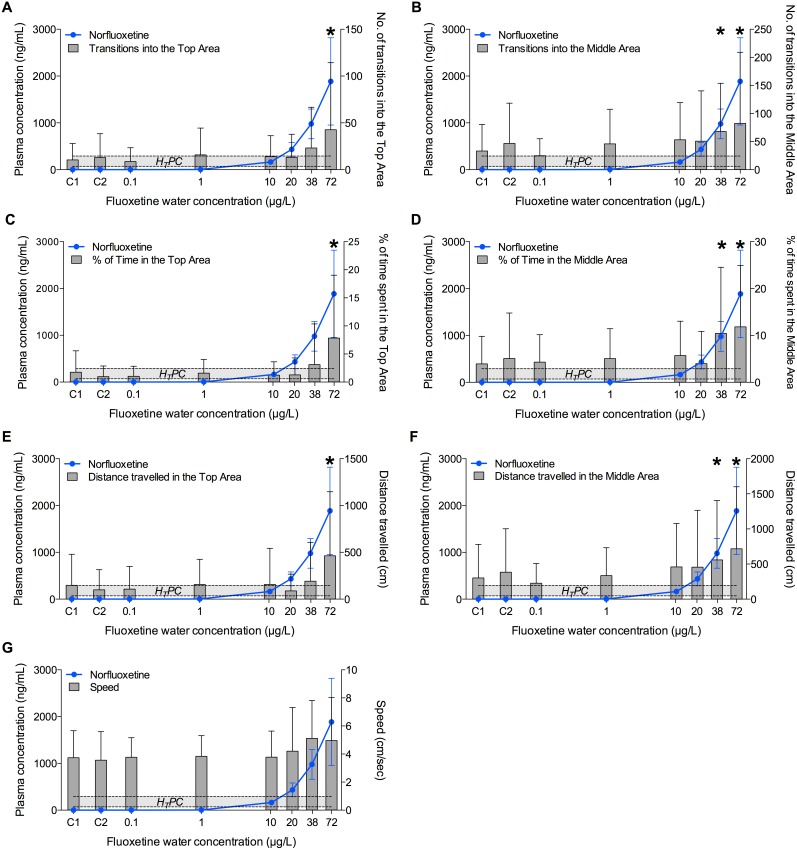
Relationship between plasma concentrations of norfluoxetine and its effects on fish exploratory behaviour after 28 days of exposure. Exploratory behaviour was quantified in individual fish using the Novel Tank Diving Test. A) Number of transitions into the Top Area; B) number of transitions into the Middle Area; C) time spent in the Top Area; D) time spent in the Middle Area; E) distance travelled in the Top Area; F) distance travelled in the Middle Area; G) speed. The Human Therapeutic Plasma Concentration range of fluoxetine plotted in the graphs is 72–258 ng/mL. C1 and C2 indicate control group 1 and control group 2, respectively. The X-axis has a Log2 scale, while the Y-axis has a linear scale. Values are plotted as mean ± SD (*n* = 20). *: *p*<0.05.

**Figure 6:**
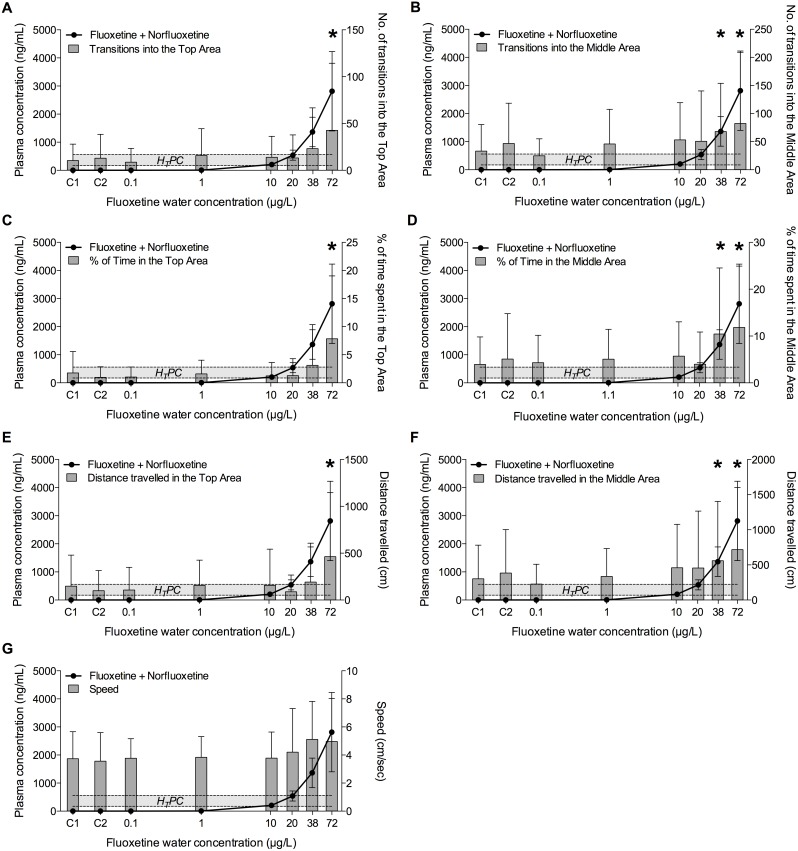
Relationship between plasma concentrations of fluoxetine plus norfluoxetine and their effects on fish exploratory behaviour after 28 days of exposure. Exploratory behaviour was quantified in individual fish using the Novel Tank Diving Test. A) Number of transitions into the Top Area; B) number of transitions into the Middle Area; C) time spent in the Top Area; D) time spent in the Middle Area; E) distance travelled in the Top Area; F) distance travelled in the Middle Area; G) speed. The Human Therapeutic Plasma Concentration range of fluoxetine + norfluoxetine plotted in the graphs is 163–560 ng/mL. C1 and C2 indicate control group 1 and control group 2, respectively. The X-axis has a Log2 scale, while the Y-axis has a linear scale. Values are plotted as mean ± SD (*n* = 20). *: *p*<0.05.

**Figure 7:**
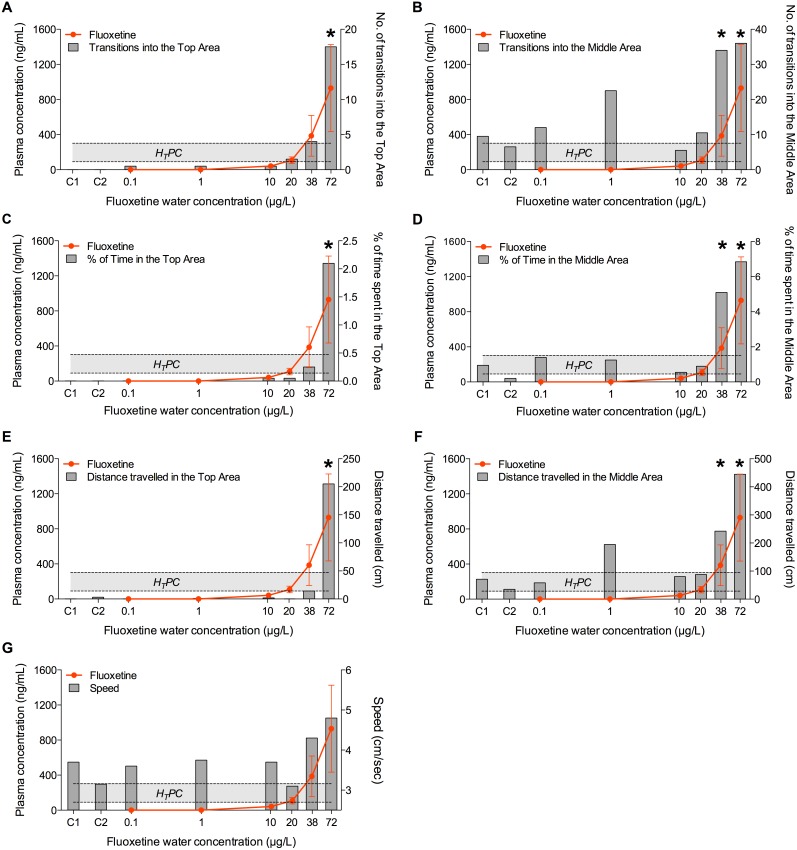
Relationship between plasma concentrations of fluoxetine and behavioural endpoints expressed as median values. Exploratory behaviour was quantified in individual fish using the Novel Tank Diving Test after 28 days of exposure. The dose-response of behavioural endpoints appears to be visually more obvious when median values are used (in this figure) instead of mean values (Figure 4). This highlights the important role of inter-individual variability in the interpretation of behavioural effects. A) Number of transitions into the Top Area; B) number of transitions into the Middle Area; C) time spent in the Top Area; D) time spent in the Middle Area; E) distance travelled in the Top Area; F) distance travelled in the Middle Area; G) speed. The Human Therapeutic Plasma Concentration range of fluoxetine plotted in the graphs is 91–302 ng/mL. C1 and C2 indicate control group 1 and control group 2, respectively. The X-axis has a Log2 scale, while the Y-axis has a linear scale. Values are plotted as medians (*n* = 20). *: *p*<0.05.

On day 28, fish exposed to the two highest concentrations of fluoxetine (38 and 72 µg/L) explored the upper areas of the novel environment more frequently and for longer time compared to the fish in the other groups, confirming the anxiolytic effects of fluoxetine in fish observed on Day 14 (Figure 4, Figure 5, Figure 6, Figure 7). Of the seven behavioural endpoints considered in this study, all except *Speed* were significantly affected in fish exposed to 72 µg fluoxetine/L (*p*<0.01 for all the affected endpoints except for the distance *Dist-Middle,* for which *p* = 0.017), whereas the exposure to 38 µg fluoxetine/L significantly affected only the endpoints related to the Middle Area (*T-Middle*, *p* = 0.03; *Time-Middle*, *p* = 0.013; *Dist-Middle*, *p* = 0.016) (Figure 4). These results suggest that 38 µg fluoxetine/L induced the fish to swim more frequently in the Middle Area, but this effect was not strong enough to induce fish to swim in the Top Area of the observation tank (the furthest from the “safe” bottom area).

Both in the control and in the unaffected treatment groups (0.1, 1, 10, 20 µg/L) a high intra-group variability in the behavioural responses was observed. A more detailed analysis revealed that that variability was due to the presence of a few very active fish in each group (i.e. dominant fish), including in the control. For example, fish in control groups (C1 and C2) were often characterized by “zero” values in all the endpoints related to the exploration of the Top Area. On the other hand, the active fish, independently from the treatment with fluoxetine, had very high numeric values for the same endpoints. This implied that, when mean values were considered, the very high values of the active fish were “spread” among the zero values of the inactive fish, resulting in the apparent variability. This effect became even clearer when median values were used instead of means, as showed in Figure 7. This type of data visualization showed a clear dose-response for all the endpoints related to the Top Area, whereas the response of the endpoints related to the Middle Area had higher noise, including in the control groups. This suggests that the variability of those endpoints, rather than being a mathematical artefact, had an actual biological meaning, and it was possibly driven by the spatial proximity of the two areas (Bottom and Middle), which increased the chances of fish exploring them.

When the results obtained on Day 14 and on Day 28 were compared, the behavioural response of fish in the highest concentration group on Day 14 appeared to be more robust in terms of magnitude than on Day 28, in particular if the number of transitions in the Top and Middle Areas were considered. For example, T-Top was 143±127 on Day 14 but only 43±72 on Day 28. A similar difference was observed for T-Middle (200±185 *vs* 82±127). The reason for these differences may simply lie in the variability of the response at different time points. Alternatively, it is possible that fish tested on Day 14 received a lower degree of stress than the ones tested on Day 28, because, although the experimental protocol and the room used for the Novel Tank Diving Test were the same in both days, the activity of the operator in the exposure room during Day 28, which was the final sampling day (i.e. to collect fish from the exposure tanks), may have caused the observed differences in the magnitude of the response. These differences highlight the sensitivity of the behavioural responses to environmental factors, which, if not accounted for in the experimental design, may significantly affect the results obtained in behavioural tests performed on adult fish. On the other hand, 38 µg fluoxetine/L affected fish behaviour only after 28 days of treatment, whereas no response was observed after 14 days, indicating a time-dependent decrease of the LOEC.

#### Weekly behavioural observations

The analysis of the geotaxis response in fish groups revealed the high complexity of group behaviour in fathead minnow, as well as the difficulty of its interpretation. The group exploratory activity in the exposure tank depends on a number of factors that all together produced a very high inter-tank variability, even in the same treatment group. It is possible to hypothesise that the high hierarchic structure of fathead minnow groups, characterized by dominant/territorial and subordinate fish, was one of the main sources of the observed variability. On Day 24, in three out of four tanks in the highest concentration group (72 µg/L), fish spent between 8.7 and 12 minutes in the upper third of the tank, confirming the geotaxis response also when fish were kept in groups. However, also fish in two replicate tanks in the control group C1 spent, respectively, 9.7 and 10 minutes in the top area. This result suggested that habituation to the tank environment occurred, so that with the progress of the study some groups tended to explore more the upper areas of the tank independently from the presence of a chemical in the water. This natural behaviour overlapped with fluoxetine-induced behaviour, complicating any reliable analysis of the association between fluoxetine exposure and the observed response. Once again, this observation may be linked to the development of dominance in the fish group. For example, in some cases the dominant fish was aggressively controlling the bottom of the tank, and this behaviour induced the remaining fish to swim in the upper areas.

Our results refer specifically to the visual analysis of fathead minnow group behaviour. However, the use of alternative fish species with weaker social hierarchies (e.g. zebrafish), together with the use of specific software able to track multiple animals (e.g. Zebralab, Viewpoint), may represent a successful solution to the problem of the quantification of the effects of chemicals on group behaviour.

### Validation of the Read-Across Hypothesis

In this study, significant effects on the exploratory behaviour in a novel environment were observed only in fish with plasma concentrations of fluoxetine approximately equal to the highest value of the fluoxetine H_T_PC range (1.3±0.8 fold) or higher (3±1.7 fold). When the sum of fluoxetine and norfluoxetine was considered, the previous values increase (2.4±0.9 fold and 5±2.5 fold respectively) above the highest value of the H_T_PC range. These results indicate that the minimum plasma concentrations of active metabolites required to elicit an anxiolytic response in fish after 28 day of exposure to fluoxetine were close to the upper value of the H_T_PC range. No effects in these fish were observed at plasma concentration below the equivalent H_T_PCs. The relationship between behavioural endpoints and plasma concentrations of fluoxetine, norfluoxetine and fluoxetine+norfluoxetine are shown, respectively, in Figure 4, Figure 5, and Figure 6.

## Discussion

Estimating the sensitivity of model species to human pharmaceuticals is a fundamental aspect of pharmacology and toxicology. To determine the sensitivity of fish to fluoxetine we investigated the equivalence of the pharmacodynamic response between fish and humans by testing experimentally the so called “Read-Across Hypothesis” [11]. The question we tried to answer was whether the same plasma concentrations of fluoxetine in both fish and humans produce comparable target-mediated effects at similar levels of biological organization, and if these effects occur only at plasma concentration equal or higher than those that are effective in humans. Our experiment successfully validated the Read-Across Hypothesis, and demonstrated that fluoxetine elicits anxiolytic responses in fish only when its plasma levels were proximate to the higher values of human therapeutic plasma concentration range, or higher.

### Relationship between behavioural effects and drug plasma concentrations

In humans, fluoxetine acts by inhibiting the serotonin transporter (SERT) in the pre-synaptic cell, which in turn leads to increased extracellular levels of serotonin in the synaptic cleft and to the consequent activation of post-synaptic receptors [32]. The overall clinical effect of elevated mood and decreased anxiety is thought to be due to adaptive changes in neuronal function, resulting in enhanced serotonergic neurotransmission. In fish, SERT is highly conserved and shows 69% identity to the human protein (94% if the aminoacids with known function in SSRIs binding are considered) [20]. From the pharmacodynamics standpoint, Gould and collaborators tested the ability of several SSRIs to displace [^3^H]-citalopram bound to fathead minnow SERT *in* *vitro*, reporting a *k_i_* value for fluoxetine in the low nanomolar range (11±5 nM) [33], which is not too dissimilar from the *k_i_* values reported in a similar assay by Owens *et al.* [34] for rat cortex (2±0.1 nM) and human transfected cells (0.9±0.06 nM). These data allow us to hypothesise that similar fluoxetine concentrations in the plasma (or at the target level) may cause comparable pharmacodynamic response in both fish and humans, leading to similar phenotypic responses at similar levels of biological organization (i.e. anxiolytic behaviour).

Extrapolating behavioural effects between fish and humans may appear an overambitious challenge for the cross-species extrapolation, given the high complexity of human behaviour, and the unquantifiable role of social factors in the response to the drug. However, fish models and particularly zebrafish have acquired a central role as model species for studying the molecular mechanisms of behavioural pathologies, due to the high evolutionary conservation of the neurotransmitter-related systems [35]–[39]. The observation of complex stereotypical patterns in fish behaviour, combined with the knowledge of the above-mentioned systems, allowed the pharmacological dissection zebrafish behavioural responses and is currently leading to an insightful characterization of many chemical-induced behavioural responses in fish (e.g. photomotor response, rest/wake behaviour, acoustic startle, habituation) [40]–[45].

In our study, we focused our attention on an anxiety-related behaviour of adult fish, which we selected as the most appropriate endpoint to test and compare Mode-of-Action (MoA) driven effects between fish and humans [21]. Fluoxetine-induced anxiolytic responses in fish were hypothesised as functionally equivalent to the reduction of anxiety in human patients following fluoxetine treatment. Fluoxetine exposure elicited anxiolytic responses in fathead minnow, and the highest concentration (72 µg/L) induced fish to visit more frequently and for more time the upper part of the tank, whereas half of that concentration, interestingly, enhanced fish exploratory behaviour only up to the middle area of the tank, demonstrating that the geotaxis response is dose-dependent. The lowest concentration that induced a significant behavioural response was 38 µg/L, corresponding to plasma concentrations of fluoxetine and fluoxetine+norfluoxetine, respectively, 1.3- fold and 2.4-fold above the highest value of the H_T_PC range. Several studies have demonstrated that fish respond in a consistent way to both anxiolytic and anxiogenic compounds [21], [22], [26], [46], [47], and exposure of zebrafish to 100 µg fluoxetine/L for 7 days is generally used as the standard treatment in studies comparing the potency of chemicals in inducing anxiolytic or anxiogenic effects [25]. This concentration increased the time spent by zebrafish in the upper part of the tank and the number of transitions into the top area [25], [26], complementing the results obtained in our study with fathead minnow. Notably, neither the total distance travelled nor the swimming speed were affected by fluoxetine both in our study and in others performed on zebrafish [26]. The anxiolytic responses observed in fish are also in full agreement with the anxiolytic effects caused by fluoxetine treatment in rodents [48], [49] and humans [23], [24], confirming the high potentiality of fish models in translational biomedical sciences.

An important aspect to consider for the determination of species sensitivity to pharmaceuticals is the comparison of healthy animals with humans affected by pathological conditions (e.g. clinical depression). This difference should be taken into account since the “molecular landscape” of an individual with a pathological condition will probably be different from that of a healthy one, and this may change the sensitivity of a given endpoint to the same concentration of drug. In this regard, a study performed by Wong and collaborators [22] investigated the effects of a 2-week fluoxetine exposure treatment on a specific strain of zebrafish, the “High Stationary Behaviour” (HSB) line. This line is characterized by high levels of stress and anxiety-related behaviours across multiple assays, including the Novel Tank Diving test, in which it shows high stationary behaviour at the bottom of the tank, and therefore less pronounced exploratory behaviour of the top area [50]. The results showed that a concentration of 11 µg fluoxetine/L was sufficient to significantly increase the time spent in the top half of the tank during the Novel Tank Diving Test, suggesting that “anxious” zebrafish are more sensitive to fluoxetine than the “healthy” fathead minnows used in our study (LOEC: 11 µg/L vs 72 µg/L). This difference in sensitivity, which could be drug-specific and species-specific, may have important implications for the use of fish in pharmacological studies, as well as for the risk assessment of pharmaceuticals in the environment, since it implies that, from a Read-Across standpoint, healthy fish may be less sensitive than mammal models or humans affected by pathological conditions.

Both in fish and humans treated with fluoxetine it is possible to identify responders and non-responders. In our study, the four replicate fish groups could be divided into high-responders, medium-responders and non-responders. Similarly to studies performed in humans, no differences in drug plasma concentration were observed among responders and non-responders [51]. The explanation for the non-response are still unclear, and may well be the results of a combination of factors including genetic polymorphisms, subject-specific molecular etiology of the behavioural disorder, and inter-individual variability in neurophysiology and sensitivity to the treatment.

One of the main clinical challenges of SSRIs is the difficulty in establishing which plasma concentrations produce the optimal balance between antidepressant efficacy and tolerability. Several studies have attempted this characterisation for fluoxetine, but without success [51]–[53]. A similar difficulty in defining a clear dose-response has been encountered for many other anti-depressants, including citalopram [54], paroxetine [55], and sertraline [56]. As Preskorn pointed out [32], one of the reasons for these results could be the “signal-to-noise” problem created by placebo responses, combined with the fact that those studies used doses equal to or above the effective minimum dose, and therefore examining only the plateau of the dose-response curve, where no dose-response is expected. On the other hand, pharmacological endpoints (e.g. serotonin uptake inhibition) clearly demonstrate the concentration-dependent effects of SSRIs [32]. This discrepancy in the observation of the dose-response may be due to several reasons, including the “noise” created by self-consciousness and social factors. In this context, it possible to argue that, unlike humans, fish do not possess self-consciousness and therefore placebo effects do not exist in this model. This implies a reduction of the “noise” of the behavioural response, allowing a sharper characterisation of the dose-response compared to human studies. Indeed, our results demonstrated that fluoxetine induced a concentration-related response in complex behaviour (i.e. anxiety) only at concentrations similar or higher the effective H_T_PCs, with no response at concentrations below the H_T_PCs, thus confirming the parallelism between pharmacological and behavioural dose-response.

### Fluoxetine uptake and metabolism

Fluoxetine was metabolized to the equipotent metabolite, norfluoxetine, in fish, as it is humans. In mammals, several *in* *vitro* and *in* *vivo* studies have demonstrated that fluoxetine metabolism involves several P450 (CYP) isoenzymes, including CYP2D6, CYP2C9, and CYP3A4 [57]. Among these, no orthologs to CYP2D6 and CYP2C9 have been identified in fathead minnow or zebrafish, whereas CYP3A4 is highly conserved in both species (92% and 94% of sequence identity to the human protein, respectively) [58]. The difficulty in identifying orthologs for the genes of the CYP2 family in fish was also confirmed by Goldstone [59], who observed that when CYP genes involved in the metabolism of xenobiotics are considered, inferring the relationships between zebrafish and human genes is difficult, mainly because of the whole genome duplication that occurred in teleosts, and of the uneven gene duplication and gene loss that has occurred in different species and gene lineages. This scenario highlights the challenge in predicting the metabolism of human pharmaceuticals in fish using the concept of conservation of molecular pathways. In the case of fluoxetine, the enzymes responsible for its metabolism in fish are yet to be characterized, and in the context of the read-across, this implies that empirical observations of the differences between the two species are the only comparative option available at present.

Fluoxetine concentrations in fish plasma followed bi-phasic kinetics. Plasma concentrations increased linearly between water concentrations of 0.1 and 20 µg/L; however, between 20 and 72 µg/L the increase of fluoxetine in the plasma, though still linear, was characterized by a sharp change in the slope of the curve. Notably, the concentration of fluoxetine at which the slope changed corresponded exactly to the decrease of the ratio NFLX:FLX, indicating that at plasma concentrations of approximately 100 ng/mL, the saturation and inhibitory effects of fluoxetine on the enzymatic system that mediate its metabolism started to play a major role in the pharmacokinetics. This effect is also extensively documented in both humans and rodents [48], [60], [61] and, although the identity of the enzymes converting fluoxetine into norlfuoxetine is still unknown in fish, the similarity of the saturation dynamics to the mammalian ones is striking. Indeed, among SSRIs, fluoxetine was one of the strongest inhibitors of human CYP2D6 *in* *vivo* [62], [63]. This inhibition was observed after daily administration of 20 mg fluoxetine for 3 weeks, which corresponded to a plasma concentration of approximately 47 ng/mL. This value is, interestingly, close to the range of fluoxetine concentration in fish plasma (40–100 ng/mL) at which the inhibitory/saturation effects started to be observable, suggesting that plasma concentrations of drug able to inhibit and or saturate CYP2D6 (or its fish counterpart) may be similar in both humans and fish.

Amsterdam and collaborators [51] measured the plasma concentrations of fluoxetine and norfluoxetine in 615 patients treated with 20 mg fluoxetine/day for 8 weeks, and observed a FLX:NFLX ratio close to 1.0 (0.9±0.7), indicating that the levels of circulating norfluoxetine are slightly higher than those of fluoxetine. Our results indicated that fish can convert fluoxetine into norfluoxetine in a more efficient way than humans, as shown by the higher NFLX:FLX ratio. This may be due to a different level of expression of the genes coding for the enzyme able to metabolise fluoxetine or, alternatively, to different genetic polymorphisms than the human counterpart.

In our study, we documented a significant inter-individual variability in the plasma concentrations of fluoxetine and norfluoxetine in fish exposed in the same tank to the same concentration of drug (up to 7.6-fold for fluoxetine, and up to 4-fold for norfluoxetine). As in fish, several studies reported a significantly high inter-individual variability also in humans, generally between 3- and 7-fold [64]–[66]. One of the possible explanations for this variability may be the genetic polymorphisms that can affect the catalytic activity of the CYP enzymes, leading to the co-existence of poor metabolisers and ultra-rapid metabolisers within the same population [57], [67]–[69]. It is therefore possible to infer that individuals with different degree of metabolic capacity may be present in fish, as they are in humans.

### Accuracy and applicability of the Fish Plasma Model

One of the major methodological challenges of this study was to select water concentrations of fluoxetine able to produce, after 28 days of exposure, plasma concentrations of the drug that were respectively, below, equivalent, and above the H_T_PCs range. This critical step for testing the Read-Across Hypothesis was successfully achieved by applying the Fish Plasma Model. Only one other study [70] has, to our knowledge, attempted to link plasma concentrations to therapeutically-relevant effects in a laboratory study using fathead minnow and the SSRI sertraline. In that study, however, all the tested water concentrations produced plasma levels above the H_T_PC, hence the Read-Across Hypothesis could not be fully tested.

In our study, the FPM generated highly accurate predictions for the group exposed to 10 µg fluoxetine/L (Δ_measured-predicted_ = 14%). The difference between predicted and measured concentrations started increasing significantly in the group exposed to 20 µg fluoxetine/L, in which measured values differed from the predicted ones by 32%. However, if the intra-group variability of plasma concentrations is considered (up to 3.5-fold), the FPM can still be considered highly accurate.

This result confirmed the high potential of this simple steady-state model to predict the bio-concentrations of pharmaceuticals in fish plasma; however, a few notes of caution need to be provided. Firstly, the accuracy of the prediction seems to be intimately related to the accuracy of the estimation of the drug partitioning factor (i.e. LogD_7.4_). Since experimental values are not always available, the potential inaccuracy in the prediction of the partitioning factor is reflected in the output of the FPM, and further amplified by the logarithmic scale of the LogD values. Secondly, considering the short-term duration of many exposure studies performed on fish in laboratory set-ups, the actual steady-state nature of the FPM will need to be carefully evaluated. In this context it may be useful to compare the output of the FPM with those of a physiologically-based-pharmacokinetics (PBPK) model for the same compound, in order to better investigate the time-dependency of the accumulation and the steady-state dynamics. Finally, it is important to consider that the FPM is based on the physiology of adult fish, therefore future research should also address developing predictive models for the uptake of drugs in fish embryos and larvae, which are highly sensitive life stages. Understanding the factors driving the uptake of a drug into fish plasma via the gills will provide additional clarification about the reliability of the model, and help to define its domain of applicability. Nevertheless, the FPM remains, at the moment, a sensible starting point to guide the design of powerful experimental designs by maximising the existing knowledge of the pharmacology of the test compound.

### Implications for the Environmental Risk Assessment of pharmaceuticals

The concentration of pharmaceuticals in environmental matrixes (e.g. water) has been so far one of the key factors in the ERA process. However, environmental concentrations are only one aspect, since it is the internal concentration of a drug (e.g. in the blood or in a target tissue) that ultimately induces pharmacological or toxicological responses in the organisms. Dramatic differences can be observed in the uptake profile of pharmaceuticals present in the water at the same concentration, even between different chemical forms of the same parent compound, as is well exemplified by glucocorticoids [11]. Understanding the factors that affect uptake is clearly crucial, as one of the key questions is whether the internal exposure levels are likely to be sufficient to induce a biological response, and if yes at what level of biological organization? In this context, the approach presented in this paper provides a new powerful tool to risk assessors by shifting the perspective from outside to inside the organism.

Our results demonstrate that the FPM can be reliably applied to predict plasma concentrations of fluoxetine in environmental realistic scenarios. Indeed, the range of water concentrations for which the model was highly accurate fully covered the environmentally relevant concentrations of fluoxetine, which are generally in the range of few tens of ng/L. For example, Oakes *et al.* [19] calculated Predicted Environmental Concentrations of fluoxetine in Surface Water (PECSW) of 0.012 µg/L, 0.010 µg/L, and 0.059 µg/L for Germany, Sweden, and United Kingdom, respectively. According to the FPM, the highest of these PEC values (0.059 µg/L) would produce, in fish, plasma concentrations approximately 1800-fold below the ones that in this study produced significant effects on fish behaviour.

More than 30 papers have reported behavioural effects of fluoxetine on fish [71]. The majority of those studies report effective concentrations between 30 and 100 µg fluoxetine/L [22], [72], or higher [73], [74]. These values can be fully explained by the results obtained in our study, which provide a pharmacological justification for the behavioural effects of fluoxetine on fish reported in the literature.

Given the high cost of quantifying chemicals in environmental matrixes (e.g. surface waters, effluent) and the point-like nature of a similar environmental monitoring approach (samples represent a snapshot of a very dynamic system) one could question the value when considering the effects of pharmaceuticals that may take days to reach effective concentrations. Recently hydrogeological models able to predict water concentrations of pharmaceuticals in European and US rivers have been developed and successfully applied [75], [76]. Combining this modelling approach with the use of behaviour as new endpoint for eco-toxicological studies, it would be possible to use the data obtained in our study and the FPM predictions to translate the estimates of the hydrogeological models (Predicted Environmental Concentration) into “*maps of risk*” for fluoxetine-induced behavioural effects. In future research, the same exercise may be performed also for other pharmaceuticals whose effects on fish have been reliably characterised and for which the applicability of the FPM has been demonstrated (e.g. sertraline), and for any endpoint that is considered environmentally relevant in adult fish (e.g. reproduction, growth, immunodepression). With the increase of data availability, this approach may be also used for the risk assessment of chemical mixtures [77], thus providing a cumulative pharmacology-based risk assessment for mixtures of compounds acting with the same Mode of Action (e.g. SSRIs).

## Conclusions and Future Perspectives

Our study addressed the fundamental biological question of the pharmacodynamic equivalence between animal species and validated the Read-Across Hypothesis applied to the anti-depressant fluoxetine. We demonstrated that healthy fish responded to the drug only when its plasma concentrations reached plasma levels similar or higher than those effective in humans, which may suggest that fish are less sensitive to fluoxetine than humans. However, this apparently lower sensitivity could be at least partly explained by the plausibly different sensitivity of healthy individuals compared to pathologically affected ones, as supported by the anxiolytic effects of fluoxetine observed in experiments performed on a “anxious strain” of zebrafish [22]. In light of these results, the overall sensitivity of fish to fluoxetine is not so dissimilar from that of patients affected, for example, by general anxiety disorders. Moreover, these results highlight the importance of using the right model in different extrapolation scenarios. For example, whilst healthy fish are appropriate in the ERA context, disease models may be more suitable for refined pre-clinical safety applications.

We demonstrated that anchoring the interpretation of pharmacological and toxicological effects to the internal concentrations of pharmaceuticals provides a quantitative benchmark to perform qCSE. Considering the importance of this aspect, future research should be addressed at improving our understanding of absorption, distribution, metabolism, and excretion (ADME) processes in fish in order to enhance the translational power of the qCSE. The validation of the Read-Across Hypothesis also for other pharmaceuticals may foster the use of fish models in toxicological and pharmacological disciplines, and thanks to its quantitative features, the Read-Across approach could be easily integrated with the recently proposed Adverse Outcome Pathway approach [78] to develop a powerful pharmacology-informed tool for the prediction of pharmacological and toxicological effects in fish models.

## Supporting Information

Table S1 **Liquid chromatography method for the separation of fluoxetine and norfluoxetine in water and fish plasma samples.**
(DOCX)

Table S2 **Mass spectrometry method for the separation of fluoxetine and norfluoxetine in water and fish plasma samples.**
(DOCX)
