# Supplementary material for: Quantitative Cross-Species Extrapolation between Humans and Fish: The Case of the Anti-Depressant Fluoxetine
Source: PLoS One. 2014 Oct 22;9(10):e110467. doi: 10.1371/journal.pone.0110467 (PMC4206295; doi:10.1371/journal.pone.0110467)
Supplement: Table S1 — Liquid chromatography method for the separation of fluoxetine and norfluoxetine in water and fish plasma samples. (DOCX) [file pone.0110467.s001.docx]

**Table S1.** Liquid chromatography method for the separation of fluoxetine and norfluoxetine in water and fish plasma samples.

| **Liquid chromatography** | | |
| --- | --- | --- |
| *Column* | 50 mm × 2.0 mm (id) | |
| *Column packing* | Gemini-NX C18 (3.0 µm) | |
| *Column temperature* | 50 °C | |
| *Injection volume* | 20 μl | |
| *Effluent flow rate* | 500 µl/min | |
| *Eluent A* | LCMS grade 0.1% ammonium hydroxide in water | |
| *Eluent B* | LCMS grade 0.1% ammonium hydroxide in methanol | |
| *Wash solvent* | HPLC grade 0.1% formic acid in 90/10 methanol/water | |
| *Gradient* | | |
| *Time (min)* | *A (%)* | *B (%)* |
| 0 | 90 | 10 |
| 4.0 | 0 | 100 |
| 6.0 | 0 | 100 |
| 6.1 | 90 | 10 |
| 8.0 | 90 | 10 |
