# Supplementary material for: Quantitative Cross-Species Extrapolation between Humans and Fish: The Case of the Anti-Depressant Fluoxetine
Source: PLoS One. 2014 Oct 22;9(10):e110467. doi: 10.1371/journal.pone.0110467 (PMC4206295; doi:10.1371/journal.pone.0110467)
Supplement: Table S2 — Mass spectrometry method for the separation of fluoxetine and norfluoxetine in water and fish plasma samples. (DOCX) [file pone.0110467.s002.docx]

**Table S2.** Mass spectrometry method for the separation of fluoxetine and norfluoxetine in water and fish plasma samples.

| **Mass spectrometry** | | | | |
| --- | --- | --- | --- | --- |
| *Source type* | Heated electrospray ionization | | | |
| *Polarity* | Positive | | | |
| *Sheat gas pressure* | 50 (N2) | | | |
| *Auxiliary gas pressure* | 20 (N2) | | | |
| *Ion sweep gas pressure* | 0 (N2) | | | |
| *Spray voltage* | 3.0 kV | | | |
| *Capillary temperature* | 300 ºC | | | |
| *Vaporizer temperature* | 300 ºC | | | |
| *Capillary offset* | 30 V | | | |
| *Q2 gas pressure* | 1.0 mTorr | | | |
| *Compound* | **Fluoxetine** | **Norfluoxetine** | **Fluoxetine-d5** | **Norfluoxetine-d5** |
| *Precursor Ion m/z* | 310.1 | 296.0 | 315.1 | 301.0 |
| *Product Ion m/z* | 44.7 | 134.1 | 44.7 | 139.1 |
| *Scan Width m/z* | 0.50 | | | |
| *Scan Time m/z* | 0.1 s | | | |
| *Collision Energy* | 12 | | | |
| *Tube lens* | 99 | 90 | 100 | 99 |
